# Hospital and Institutional News

**Published:** 1912-09-21

**Authors:** 


### HOSPITAL AND INSTITUTIONAL NEWS.

### THE BRITISH HOSPITALS ASSOCIATION.

Almost immediately after we go to press this week the third annual conference of the British Hospitals Association will have begun. It is very much to be hoped that a good rally of hospital officers will be present, as the programme, which we published last week, promises some very interesting discussions. The papers generally, indeed, cover a wide field, and combine very happily the high politics of hospital work with a treatment in greater detail of subsidiary but no less important matters. The paper of Sir William Collins, with which the conference opens, deals with the great question of the relation of "Hospitals to the State" in a comprehensive spirit, and from a widely considered, not to say historical, point of view. He will be followed, as readers of the programme will remember, by Mr. Edgar S. Kemp on the "Training and Work of the Almoner." Thus, on the first day the hospital statesman and the hospital officer will each have plenty to occupy his mind. In the afternoon the Insurance Act will occupy the attention of the conference, Dr. Nathan Raw and Mr. Basil Rhodes contributing papers on this subject, to another aspect of which we return in our leading article this week. On Friday Mr. Danvers Power will read a paper on "Hospital Management considered in Relation to Responsibility," while the afternoon will be devoted to visiting the hospitals of the city. We recall these facts for the benefit of those who may have been prevented from attending; but as there were many regrets expressed last year after the public interest in the Manchester Conference had become common property, so we are sure that those who have not attended this year will look back with regret next week when they are able to peruse the report of the proceedings in our own columns. By the time this issue is in the hands of our readers it will be too late to attend the discussions at Birmingham, but it will not be too late to join the British Hospitals Association, whose sphere is unique and whose record of work done for the voluntary hospitals is already considerable and a great credit to its organisers, in particular to Dr. Mackintosh, M.V.O., its energetic honorary secretary, and to Mr. Conrad Thies and Mr. Stewart Johnson, its hon. treasurers, to either of whom applications for membership should be sent, at the Western Infirmary, Glasgow, or at the Hospital for Sick Children, Great Ormond Street, London, W.C.

### A SUFFRAGETTE LADY DOCTOR AND THE INCOME TAX.

Amusing events are in progress at Upper Clapton concerning a well-known lady practitioner, Dr. Wilks, who resides in the place with her husband, himself a London County Council schoolmaster; as one of the founders of the Suffragettes' Tax Resistance League, Dr. Wilks has refused to pay income tax. The Inland Revenue authorities have been in lengthy correspondence with Mr. Wilks, but to no purpose, and we understand that when two officers arrived with a warrant for his arrest they were informed he was ill in bed; he is told that arrest awaits him immediately he leaves the house, but even in the face of this fact Dr. Wilks refuses to relinquish her position of defiance. The Inland Revenue authorities are understood to have proposed to distrain on her furniture, but Dr. Wilks has intimated that if her furniture were sold she would bring an action for damages for illegal execution. It will be remembered that some time ago Mr. Bernard Shaw pointed out that no power could compel a wife to reveal the amount of her income to her husband for purposes of income-tax assessment, and that he was helpless if she refused to do so.

### GENERAL NOGI AND LONDON HOSPITALS.

The dramatic death of General Nogi has focussed public attention upon the career and life history of this great Japanese commander. Few of the obituary notices, excellent as they are, have, however, laid stress on the fact that the late general was an active supporter and cordial friend of the Japanese hospitals, and that his interest in hospital work extended beyond Japan. During his recent visit to London he privately and entirely unostentatiously visited one of the big Metropolitan hospitals, and in America he visited more than one institution. In Japan he was a frequent visitor to the wards of both the military and civil hospitals, and he showed his active interest in hospital extension by promoting and subscribing to the extension of the well-known pathological block at Tokio. During the two wars in which he played such an important part he won, abroad at all events, the reputation of being a reckless, dare-devil commander, who needlessly sacrificed life to attain victory. Those who know the painstaking care with which he superintended the organisation of the Japanese Red Cross Army Service, and the manner in which he helped and furthered the work of this hard-pressed department, are not likely to subscribe to this popular view.

### THE STAFF'S POLICY AT SWANSEA HOSPITAL.

The action of the honorary medical staff of Swansea Hospital, every member of which, with one exception, has signed the pledge of the British Medical Association, has been further considered by the committee of management. The committee has decided to defer action till November 27, but the meeting at which this decision was arrived at had an interest from the definite statement of medical policy which came from Mr. Brook, the senior member of the honorary medical staff. "Medical men," he said, "want to put a stop, and for years have been unable to put a stop, to the scandal of underpaid contract work. The very class which we are told will be affected if the action of the medical staff be carried into effect are those who (and they do not realise it themselves) have been the victims of a system which I and my colleagues regard as deplorable. The medical staff of the hospital has seen the results of underpaid contract work outside the hospital, and though their action may inflict a certain amount of hardship—it will be more inconvenience than hardship—it will be nothing compared with the evil which they are trying to bring to an end, and which the Government is seeking now not only to perpetuate, but actually to extend." That is a direct summary of the attitude which the medical staff of the Swansea Hospital has set an example of formulating with convincing clearness. It is probable that more inconvenience than hardship will result from the attitude they have taken up. This fact should be emphasised now that such wild abuse is flying about as that uttered by Mr. W. T. Hughes, a local organiser of the Dockers' Union, who described the attitude of the doctors as "Syndicalism run mad," thus making what is nothing but an economic theory for the vesting of each industry in the workers of that industry a term of abuse, and confusing it with such militant methods as the general strike, with which it has nothing necessarily in common at all. Nothing is so likely to exasperate feeling as this loose use of technical terms.

### THE NEW SECRETARY OF BOLTON INFIRMARY.

Mr. James Briscoe, the secretary of Bolton Infirmary, has now resigned after twenty-nine years in that office, being now seventy-nine years of age. His record of service is gracefully summarised in a resolution that has been passed by the committee. We recall, the resolution remarks, "how, in a past now grown somewhat distant, he was one of the founders of the Hospital Saturday Committee, of which he became hon. secretary, and as a member of that association he became a member of the infirmary committee when the hospital was still in its old home on Nelson Square. The very valuable services rendered to the infirmary by the Hospital Saturday Committee, and the agreeable relations between the two, are very largely due to Mr. Briscoe's enthusiasm and ability. In 1883 Mr. Briscoe was appointed secretary to the infirmary. In performing for twenty-nine years the duty of that office he had displayed unfailing energy, ability, industry, and resource. He has always taken a high view of his position, regarding it not merely as the performance of routine duties, but as a privilege of taking part in a noble ministry to the suffering poor." He is only retiring now for reasons of health, and after the above resolution, which was moved by Mr. W. Brimelow and seconded by Canon Chapman, had been carried, the committee appointed Mr. Albert E. Briscoe to the post of secretary in the room of his father. Mr. Briscoe, jun., has acted as assistant secretary to the Bolton Infirmary for nearly eighteen years.

### MILITARY LIFE OF A HOSPITAL SECRETARY.

As readers of The Hospital may remember, Mr. James Briscoe has also had a distinguished military career, and it is little more than a year ago that we had the pleasure to record in these columns his long-delayed receipt of the medal "granted for service in the suppression of the Indian Mutiny." He enlisted in 1854 in the 85th Durham Light Infantry, and after being made a lance-corporal he volunteered for active service in the Crimea with the 72nd Highlanders. He was present with his regiment at Sebastopol and throughout the campaign, which was followed by a period of garrison work. As the regiment was on the point of returning to Edinburgh the Indian Mutiny broke out, and Mr. (then Sergeant) Briscoe was despatched to Central India. Here his instinct for civil life also found expression in the establishment of a regimental printing office. His services were rewarded by the British and Turkish Crimea medals and six months' extra pay for field service, besides a share in the Kotah prize money. His discharge from the Service in 1865 was followed by his joining the Volunteers, and in 1879 he became paymaster-sergeant, only resigning when fifty years old in accordance with the regulations. Mr. Briscoe has also been awarded the Long Service Volunteer Medal, and it will be seen that his retirement from military life was only the prelude to a very active participation in civil, and especially in hospital, affairs.

### A CONVERTED PRIVATE WARD AS A THEATRE.

At the Johnson Hospital, Spalding, into the management of which an inquiry was held by the Charity Commissioners in the spring subsequent to our Report in The Hospital, a new theatre has been opened. The staff had made representations to the governors on the matter, for the old theatre was not, like the new one, on the same corridor as the wards, and patients had to be taken to it down a flight of curved stairs to the general and sometimes serious inconvenience of patients and doctors. The new theatre has been converted from a private ward, and two annexes, the second of which leads out of the theatre, have been employed for an anæsthetising room and a sterilising room respectively. Now, at all events, the wards and anæsthetising and operating rooms are on the same floor, which marks a great advance on the previous conditions. The hospital's president is Canon Bullock, and the honorary secretary is Mr. A. K. Maples.

### THE EXTENSION OF BRISTOL GENERAL HOSPITAL.

It is only two months since we had to record the opening of the new surgical block at the Bristol Royal Infirmary, which has been one of the most important hospital events of the year. It must not, however, be gathered from this that hospital extension has been done with in Bristol for the present, for the General Hospital is proposing to extend its borders. This will include not merely additions to the wards but to the accommodation for the resident staff, to the administrative department, and a new dental department is badly wanted to supplant the building where that work is now carried on at a distance from the institution. Mr. Joseph Storrs Fry, the president, pointed out at the half-yearly meeting that the majority of the committee were in favour of proceeding with the scheme notwithstanding the unknown possibilities of the Insurance Act. The extension is expected to cost about £45,000, of which all but £500 has been subscribed, and a tender has been accepted in accordance with the plans of the architect.

### CHARGES AGAINST HOSPITAL CHAPLAINS.

We wonder what hospital chaplains have to say to the letter published by the *Spectator* last week under the title "Anglican Intolerance." The writer, who signs himself "Presbyter (Wesleyan Minister)," states that the incident which he narrates happened while he was "under surgical treatment in a nursing home attached to one of the great London hospitals." The editor of the *Spectator*, rightly in our opinion, thinks that if the facts are as stated "Presbyter" made a great mistake in not appealing to the Bishop of London. The locale of the hospital we do not know, but the opening sentence of the letter implies an accusation against at least two of the best known hospitals of London (the chaplain of one of which is a bishop) and that accusation ought not to go unanswered. We have always found hospital chaplains, no matter what their doctrinal convictions were, most humane and charitable, and we hesitate to accept the charge brought by "Presbyter" as proven until its truth has been clearly shown. For that reason we think that the writer of the letter alluded to should have the courage and fairness to give our contemporary the name of the institution and of the chaplain to whom he referred. We are fully aware that mistakes are apt to occur, but the facts (if facts they are) seem to show that in this case there was no mistake in the ordinary acceptation of the word, but a deliberate intention. As to the justice of the act we offer no opinion, but it seems to us that the *Spectator* is not far wrong in stating that all true friends of the Church will be deeply grieved if the facts are as stated, and it is for the writer to clear up the charge he has brought, which, until it be settled one way or the other, will be justly resented by all hospital chaplains.

### A QUESTION BURKED BY THE BRISTOL GUARDIANS.

The hospital committee of the Bristol Board of Guardians has issued a report dealing with the situation created by the resignation of Miss Moss, assistant medical officer at Eastville Workhouse. The Rev. A. E. Bray stated that a more pitiable state of affairs, under the present arrangements, would be hard to find. When they had a male medical officer that was going to settle things. He only stayed six weeks. Then they got a lady, who had stayed long enough, but was leaving very disappointedly. The report and the majority of the Guardians, however, incline to the present state of affairs, by which an assistant medical officer is appointed under one chief medical officer, in whose control the whole of the sick, imbecile, and other patients are placed. The report was finally agreed to, it being felt that all changes should be postponed until the opening of the Southmead Infirmary, which, as already noted in these columns, will probably be accompanied by important changes in the classification of the patients and infirmaries. The new institution at Southmead will receive all the acute cases, and this will lead to the reorganisation of the medical staff. That is no reason, however, why the present unsatisfactory state of affairs should not be gone into, or why the resignations of the recently appointed medical officers should be brushed aside as of no importance.

### A "BURNING" QUESTION.

In spite of the amount which has been written on the constantly recurring fatalities resulting largely from the wearing of cheap and inflammable flannelette, voices are still to be heard, and conspicuously from the trade journals interested in the sale of this commodity, deprecating legislative interference. Thus the *Draper*, while piously welcoming any legislation which would "effectually prevent the terrible annual loss of infant life through burning accidents," takes Dr. Waldo, the City coroner, to task for thinking that "this desirable end can be obtained by restrictions and regulations on the sale of flannelette." So much for the sincerity of its "welcome"! The journal then goes on to suggest "that legislation should be passed making it punishable for any mother to leave children below a certain age with an unguarded fire." This has been done already, as the *Draper* would see by turning to a clause in the Children Act of 1908, which, largely thanks to pressure from the coroners, for the first time makes it a punishable offence for a parent or guardian to leave a child under seven years of age alone in a room with a fire burning in an open grate unguarded or insufficiently protected, the guardian being over sixteen years of age. What the Legislature must further do to complete its protective action in this matter is to insert a clause in the Merchandise Marks Act making it also a punishable offence to publish a false description of flannelette, such as "safe," when after a few washings its non-inflammable qualities disappear. Now that the Legislature has long since adopted our contemporary's suggestion, where its judgment was unbiassed, it will be admitted generally that others may be better judges of further legislation which affects its trade interests.

### SUNDAY WORK IN WORKING MEN'S CLUBS.

In recognition of his work as secretary of the Beckett Hospital Saturday and Sunday Hospital Committee, and of his association of thirty years with local hospital work, a presentation was made last week to Mr. Alfred Whitham in the Barnsley Town Hall. Alderman J. S. Rose presided, and reminded the meeting that he had worked side by side with Mr. Whitham, who had put his heart and soul into the movement which had made the Beckett Hospital one of the best institutions outside London. In making the presentation, the Mayor, Councillor J. H. Cotterill, said Mr. Whitham was a many-sided man, and had been for many years a member of the Corporation and chairman of the sanitary committee. When he first began to organise the Saturday and Sunday movement Mr. Whitham had to do all the work that was now done by the various village committees. It was he who made arrangements in the outside districts. The presentation included a purse of gold and a coffee service, the coffee pot having the words: "Presented to Mr. Alfred Whitham for long and valuable services to the Beckett Hospital, 11th September, 1912." In the course of his reply, Mr. Whitham said he was merely retiring from the active work of secretary: the work required a younger man, and he believed his successor, Councillor Tipping, would spare no effort to make it a big success. He still represented the working men on the house committee, he had been elected deputy chairman of the hospital, and he presided at meetings in the absence of Mr. Bond. He referred to the multifarious duties of a hospital secretary, which gave a secretary but little time at home on Sunday, he often having to be at three places on the same day. He had many pleasant recollections to look back upon, and he rarely missed visiting the hospital every day. In thanking the Mayor for presiding, Alderman Rose remarked that Mr. W. W. Smith, the first secretary of the Saturday and Sunday Hospital Committee, was present, and that their first effort realised £100, and now they averaged £2,300 a year. Mr. Whitham's most valuable service had been perhaps the reorganisation which he had introduced. The proceedings concluded with a suggestion from County Councillor Plumpton that the hospital committee should pay more attention to Sunday work, by addressing the men's clubs, at which he had known two hundred men to be present, who were glad to be addressed and gave liberally to the collection which followed.

### THE EXAMPLE OF THE MONYHULL COLONY.

According to the latest annual report of the Local Government Board, Poor-Law Guardians generally are awake to the importance of removing epileptics, imbeciles, and the feeble-minded from the workhouses. It seems likely, in fact, that combinations of Unions will be formed for this purpose, though the expense of providing the necessary accommodation is a serious difficulty, which some propose to leave for the county authorities. As a model of what may be done in regard to epileptics at any rate, the Monyhull Colony, near Birmingham, stands now before us. The establishment is the result of three Boards of Guardians co-operating, and 252 patients are accommodated. The surroundings in which the treatment is carried on, the amount of useful work which the patients, or, as they are usually called, the colonists, perform is a revelation of what can be done when the three principles of co-operation between Unions, carefully chosen surroundings, and productive treatment are put into force. So successful, in fact, has the colony proved that the joint committee of management is considering the possibility of extension for the purpose of providing accommodation for a number of children. Not the least valuable feature of the Local Government Board's annual report is the lessons which it deduces from the unqualified success of the Monyhull Colony.

### CONSUMPTION AND HOUSE INSPECTION.

Local schemes for the treatment of tuberculosis are now of mushroom growth, and sometimes rivals, as the following example will show. The Surrey County Council has prepared a scheme of sanatorium or dispensary treatment, but this is now sharply criticised by the Medical Officer of Health to the Richmond Town Council, Dr. Crocker, who asserts that Richmond can achieve the same results at half the projected expenditure. He charges the County scheme with overlapping and division of responsibility, and contends that house-to-house inspection, with the prevention of overcrowding that may be said to result from it, will produce far better results than "tinkering with the individual," whether at the dispensary or at the sanatorium. His arguments have so impressed the Council that they have decided to ask the Local Government Board to defer the passing of the County scheme in so far as Richmond is affected by it. There can be little doubt that Dr. Crocker is on the right lines in his commendation of general preventive measures, but he must supplement them with a dispensary (the two agencies should be complementary) to act as a sifting centre, to provide treatment for incipient cases, and to educate the locality. Probably it was his arguments against expending vast sums of money on sanatoria that awakened the sympathy of the Council. Prevention and treatment must begin in the home; but the problem is not to be solved by house-to-house inspection only.

### A MEDICAL SKIT ON FORCIBLE FEEDING.

Those of us blessed with a sense of humour should feel grateful to Dr. Mercier for his timely exposure of the absurdity of the recent "Preliminary Report on Forcible Feeding." Dr. Mercier's parody appears in last week's *Lancet*, and is one of the best examples of what Newman called "convincing ridicule" that we have read. He follows the style and the bad grammar of the notorious report with a pertinacity that is worthy almost of a more serious subject. The skit must be read in its entirety in justice both to the delicacy of the satire and the grotesqueness of the report satirised, but we may allow ourselves one quotation. "To the physical torture of forcible bathing the Home Secretary adds in many cases the intellectual torture of rubbing with bath towels, often made of huckaback. After the terrible ordeal of the bath the prisoner often fell into a state of collapse, from which he was aroused by the hideous uproar of the next bathing time. The sickening thud of bodies thrown from upper windows, the crash of breaking bones, the ferocious oaths of the prison medical officers, the shouts of diabolical laughter of the warders, the hymns from the tortured prisoners . . . driven mad by the human barbarity of a fiendish Home Secretary, together make up a pandemonium that might well shake the nerves of the strongest." Somebody would do a kindness by presenting the three signatories with a strongly bound copy of Dr. Mercier's well-known text book on Logic.

### CONSUMPTIVES IN ENGLISH HOTELS.

The public health committee of the Essex County Council, subject to the approval of the Local Government Board, have appointed three practitioners with special experience of the treatment of tuberculosis to act as tuberculosis officers under a temporary dispensary scheme for the county. The salary is £500 a year each, and there were sixty-eight applicants. As part of what is called the anti-phthisis campaign and the growing popularity of the dispensary as the centre of operations, these appointments have a certain interest. But, while everyone is noting the medical and administrative efforts now being made for the general arresting of the disease, few pause to inquire what effects such activity is producing on the general mass of the public. One of them is worth recording: it is a greatly increased and exaggerated fear of contracting this disease, such as a few years ago was unknown in this country. Even so lately as five years ago the average Englishman was surprised and a little disgusted at the way in which consumptives were treated like lepers in hotels on the Continent, and at the morbid horror of illness which expressed itself in forbidding even perfectly healthy people to sit in long chairs on their balconies. To-day, thanks, no doubt, to a misunderstanding of the anti-phthisis propaganda, the English hotels are following suit, and Torquay and such places which used to advertise as winter resorts for people in weak health now spurn from their doors anyone who even hints at a sickness or coughing. May the newly appointed county tuberculosis officers exorcise from the lower middle-classes this morbid and ridiculous spirit of alarm!

### THE MAKING OF THE MENTAL HOSPITAL.

Dr. Joseph Shaw Bolton, medical superintendent of the West Riding Mental Hospital, Wakefield, has several interesting things to say in his report, among them some of the causes which hinder the mental hospital, when judged by results, from being regarded as an institution for the cure of mental disease. Taking his list of admissions, he finds a total of 274 probably or entirely incurable, with 193 possibly or probably recoverable, and he goes on to say that such figures point to the need of legislative measures to arrest the mental deterioration of the race, by extending the preventive work which mental hospitals are now able to perform. He regrets that segregation has been the only general measure adopted. At his own institution, as is increasingly the case and well instanced in our recent account of the improved diet and service at the Leicester Borough Mental Hospital, West Humberstone, efforts have been made to improve the general health of the patients by extended out-treatment. In addition, Dr. Bolton hails with natural pleasure the appointment as a whole-time pathologist of a professional bacteriologist of repute, Dr. Nabarro, and predicts a great future for the reorganised pathological department at the institution. His words indeed on this point deserve quotation: "The custom of appointing an unskilled junior assistant medical officer to the so-called post of pathologist is now out of date, and the time has come for the recognition of the fact that the elaborate and complicated methods of scientific medicine, from the aspects of the prevention and alleviation of disease as well as from that of research, require years of special training and experience for their acquisition." Such appointments and the demanding from assistant medical officers of the possession of a Diploma in Psychiatry, such as that lately instituted at Leeds University, are the progressive steps in the advance of the mental hospital as a curative institution, which the discarding of its former title must more and more imply.

### HOSPITAL HOUSEKEEPING AND CO-OPERATIVE FARMS.

The paper on this subject, read by Miss Armour, of the New Rochelle Hospital, New York, before the Canadian Hospital Association, is published in the current issue of the *Hospital World*—one of the most interesting and informative papers we have ever read. It appeals not merely to one class of hospital workers, and deserves to be read more especially by architects and superintendents, for Miss Armour gives valuable hints on side issues the importance of which they are often apt to overlook. For example, that ice boxes should not be connected with drainage pipes. What interests us especially in Miss Armour's paper is the excellent suggestion that every large hospital should have a co-operative farm—"somewhat along the lines of the old monasteries, which, with their appendages of fields and forges, formed the nuclei of all our great English cities." We hope shortly to publish a series of articles showing how this suggestion, which is by no means a new one, has been taken up. Meanwhile, we commend these suggestions to hospital workers, who, although they may here and there need help from an American friend to interpret certain expressions (what English superintendent knows what a "squab" is?), will find it rich in suggestion.

### A NEW INSTITUTION FOR BRIGHTON.

An important addition to the institutions of Brighton will result from the munificence of Mr. John Howard, an engineer of the town, who has given £40,000 for the erection and endowment of a convalescent home. The home is to be built just outside the eastern boundary of the borough on the sea front. On the building itself £8,000 is to be spent, which will leave £32,000 for the endowment fund. An interesting feature of the home is that it aims at accommodating two classes of patients: those who cannot afford to pay, and those who can pay moderate fees. This is only the latest example of that growth of classification which entails a sliding scale of payments from patients, and which in its fully developed form is seen in the separate pavilions for different classes which are found in so many hospitals in America.

### THE NEW COTTAGE HOSPITAL AT RIPLEY.

In the presence of Colonel Seely, Secretary for War, the new Ripley Cottage Hospital, which is built to serve the Pentrich Collieries and district, has been opened free from debt, thanks to the generosity of Mr. Charles Ford, of St. Leonards, a colliery proprietor in the neighbourhood of the hospital. Mr. Ford has already supplied the district with a public ambulance, and subscribed largely to the building fund. The hospital, which has cost £1,116, consists of four beds and a cot, all on the ground floor in both men's and women's wards, a theatre, and the matron's quarters. A notable fact about the equipment is the extent to which the women of the neighbourhood have subscribed through their religious and political associations, under the leadership of Mrs. Crossley. The workers have undertaken a voluntary contribution scheme. The chairman of the hospital is Mr. B. J. F. Crossley, and Mr. A. Wassell is the treasurer.

### SKULL COMPARISONS.

In the Anthropological Section of the British Association an interesting contribution emanated from Professor Anthony. He took as his subject the brain capacity of paleolithic skulls, and drew some very general conclusions from a comparison of the La Quina skull with the La Chapelle, Neanderthal, and other skulls. Such conclusions are largely speculative and must be of that nature since we lack the other data necessary for correlation. For our part, however, we venture to think that something useful might be gained by comparisons of fossil skulls with crania of abnormally developed moderns. It has yet to be proved that the remains found so far—in France, in Java, and elsewhere—of "prehistoric man" can be accepted as examples of types. There is just as much reason to suppose that they are the remains of aberrant individuals. The conditions under which they were preserved at least lend some support to this view. In the same Section Canon MacCulloch, well known for his researches into folk-lore generally, read a paper on "Folk Beliefs in the Highlands and Lowlands." This was one of the most interesting papers read before the Section, and it contained some points of special interest to professional men, notably in the descriptions of folk-lore medicines which are still employed. The Canon's paper is brimful of interesting points, and must be held to be one of the most popular read before the Congress.

### HONOUR FOR CRIMEAN ARMY SURGEONS.

It is fifty-eight years since the Crimean campaign drew the attention of the people of this country to the importance of an organised and equipped Medical Service for the Army. The announcement, therefore, of the reward for distinguished service to two veterans of the Army medical staff of those days cannot be regarded as premature, nor is the amount of the pension thus awarded (£50 each per annum) at all excessive. The two officers thus honoured in their old age are Deputy Surgeon-General Gulland and Deputy Surgeon-General Sinclair, C.B. The former was in the Crimea, then in the China War of 1860-2, and later on in the Hazara expedition of 1868; the latter served in the Crimea, then in the Indian Mutiny, and finally in the Boer War of 1881. In all there are some fifteen medical officers in receipt of these awards for distinguished and meritorious service, the best known of whom, perhaps, is Sir A. D. Home, V.C. The list, indeed, contains veterans whose records of service compare favourably with those of their comrades in the combatant branches, and we trust that the two gallant officers who have just been added to it will long live to enjoy both the honour and the solatium which it entails.

### HOSPITAL DISPENSERS AND THE INSURANCE ACT.

Mr. R. W. Lindsey, chairman of the Public Pharmacists' and Dispensers' Association, has been appointed a member of the Pharmaceutical Standing Committee on Insurance. The result of this appointment is that institutional dispensers are now represented on the committee. The desirability of such representation has been urged in The Hospital on more than one occasion, and it is satisfactory to know that the committee will have the advice and assistance of one who has had a large experience in institutional dispensing. It is also interesting to note that at its last meeting the committee discussed the question of the supply of drugs for sanatorium patients, and resolved to make an effort to secure that the dispensing of medicines for such patients should be done under the supervision of a qualified pharmacist.

### THIS WEEK'S DRUG MARKET.

The expected improvement in business has not yet become noticeable, and the Drug market continues rather quiet. Of price changes there have been few of importance. The position of opium is without appreciable change, prices being well maintained; morphine and codeine are also without change. Quinine, instead of advancing in price as was confidently anticipated a short time ago, is inclined towards lower prices, which seems to suggest that the attempt to bring about an agreement between growers of cinchona bark and manufacturers of quinine has again been unsuccessful. Cod-liver oil is in fair demand at the higher rates. Emetine is considerably dearer. Balsam copaiba is inclined towards lower prices, as also is oil of cubebs. Orris root is selling at very high prices. Oil of aniseed is tending higher in value. Santonin has not yet been definitely raised in price, but an announcement from the makers is daily expected.

### TO OUR READERS.

Contributions are specially invited from any of our readers to these columns. They should deal with topical subjects and news. They must be authenticated for the information of the Editor only. The minimum payment if published is 5s. There is no hard-and-fast rule as to space, but notes of about twenty lines in length are preferred.

